# It’s not only what you say, it’s also how you say it: communicating nipah virus prevention messages during an outbreak in Bangladesh

**DOI:** 10.1186/s12889-016-3416-z

**Published:** 2016-08-05

**Authors:** Shahana Parveen, M. Saiful Islam, Momtaz Begum, Mahbub-Ul Alam, Hossain M. S. Sazzad, Rebeca Sultana, Mahmudur Rahman, Emily S. Gurley, M. Jahangir Hossain, Stephen P. Luby

**Affiliations:** 1 Infectious Diseases Division, icddr,b, Dhaka, Bangladesh; 2 Institute of Epidemiology Disease Control and Research (IEDCR), Dhaka, Bangladesh; 3 Medical Research Council Unit (UK), Banjul, The Gambia; 4 Global Health Protection Division, Centers for Disease Control and Prevention (CDC), Atlanta, Georgia USA; 5 Infectious Diseases and Geographic Medicine, Stanford University, Stanford, California USA; 6 Programme for Emerging Infections, Infectious Diseases Division, icddr,b, 68, Shaheed Tajuddin Ahmed Sarani, Mohakhali, Dhaka, 1212 Bangladesh

**Keywords:** Nipah virus, Outbreak, Prevention messages, Communication strategy, Anthropological approach, Contextual understanding, Bangladesh

## Abstract

**Background:**

During a fatal Nipah virus (NiV) outbreak in Bangladesh, residents rejected biomedical explanations of NiV transmission and treatment and lost trust in the public healthcare system. Field anthropologists developed and communicated a prevention strategy to bridge the gap between the biomedical and local explanation of the outbreak.

**Methods:**

We explored residents’ beliefs and perceptions about the illness and care-seeking practices and explained prevention messages following an interactive strategy with the aid of photos showed the types of contact that can lead to NiV transmission from bats to humans by drinking raw date palm sap and from person-to-person.

**Results:**

The residents initially believed that the outbreak was caused by supernatural forces and continued drinking raw date palm sap despite messages from local health authorities to stop. Participants in community meetings stated that the initial messages did not explain that bats were the source of this virus. After our intervention, participants responded that they now understood how NiV could be transmitted and would abstain from raw sap consumption and maintain safer behaviours while caring for patients.

**Conclusions:**

During outbreaks, one-way behaviour change communication without meaningful causal explanations is unlikely to be effective. Based on the cultural context, interactive communication strategies in lay language with supporting evidence can make biomedical prevention messages credible in affected communities, even among those who initially invoke supernatural causal explanations.

**Electronic supplementary material:**

The online version of this article (doi:10.1186/s12889-016-3416-z) contains supplementary material, which is available to authorized users.

## Background

In Bangladesh, Nipah virus (NiV) has caused 189 recognized human cases of illness in several outbreaks or clusters from 2001 to 2013 with over 75 % of affected persons dying [1–7]. An outbreak is defined an increase of more cases of disease than is usually expected among a specific group of people or within a place over a particular period of time [8]. The most common pathway of NiV transmission in Bangladesh is through drinking raw date palm sap contaminated with saliva or urine of fruit bats *(Pteropus giganteus*), the natural reservoir of NiV [9–12]. In Bangladesh, sap harvesters collect raw sap from date palm trees mainly during winter (December to April) and drinking this raw sap is a local delicacy [13]. NiV can also transmit from person-to-person through close contact with respiratory and bodily secretions of NiV infected patients [2, 14].

During a NiV outbreak in 2004, qualitative investigators were first invited to join the outbreak investigation team from the Institute of Epidemiology, Disease Control and Research (IEDCR) of the Government of Bangladesh and icddr,b. Since then, qualitative investigators have been serving as core members of the collaborative outbreak investigation team [15]. They complement the epidemiological and clinical investigations by providing a contextual perspective of the outbreak [15]. The qualitative investigators follow anthropological methods to explore detailed illness histories, the behavioural and cultural factors that influence risk of transmission, causal explanations of the illness and care-seeking behaviour of affected people [14, 15].

In January and February 2010, an outbreak was recognized in Faridpur District, Bangladesh. The investigation team identified eight people infected with NiV, seven of whom died [6]. Initially, infected cases exhibited symptoms of encephalitis and/or respiratory illness. Five cases had a history of consuming raw date palm sap approximately one week before the onset of illness [6]. All but two cases sought treatment from local government hospitals. With the high fatality rate (88 %) there was panic in the affected community, similar to what was observed in a large NiV outbreak in 2004. During the 2004 outbreak, residents became suspicious about local government hospital care and of the hospital workers because of the failure of biomedical treatment to save the lives of infected patients [14]. When the 2010 NiV outbreak was identified, the local health authority used loudspeakers and household visits to tell residents that drinking raw date palm sap could cause the illness, and announced that people should stop drinking it. Despite these prevention efforts the residents continued drinking raw sap.

In this context of mistrust, it was difficult for the outbreak response team to communicate prevention messages based on the biomedical paradigm because, as in the 2004 NiV outbreak, the community understood the illness in quite different terms [14]. To prevent the further transmission of the infection, the outbreak response team wanted to build the trust with residents so they would express their beliefs, perceptions and care giving practices related to this illness, and use residents’ understanding as a basis to communicate information about NiV related to both zoonotic and person-to-person transmission. This paper describes the communication strategy we used that aimed to bridge the gap between the biomedical explanation of a highly fatal disease outbreak and the local interpretation of that illness.

### Risk communication: Traditional approach versus culture-centered approach

Traditional health communication basically presents a Eurocentric paradigm of a biomedical model of health [16]. This approach promotes understanding and explaining health problems and outcomes from a top-down approach that is often ignorant of cultural context [17], specifically in response to inherent belief within a local context, which risks undermining the effectiveness of the intervention. Health experts often describe the local perception of a disease in terms of existing spiritual beliefs and suggest that the affected population perceives the disease as originating from a supernatural cause [14] which limits their openness to scientific arguments. The logic behind these local explanations are not usually highlighted [18, 19], but are important to understand to design a culturally credible, effective intervention.

In emergency situations affected communities are often distrustful of government agencies and personnel who communicate risk messages using traditional media outlets, such as radio, television or print media [20–22]. For example, in 2000–2001 during an Ebola hemorrhagic fever outbreak in Uganda and a previous NiV outbreak in Bangladesh local perceptions, beliefs and practices were not considered during initial communication initiatives and contributed to low acceptance or rejection of the biomedical recommendations for disease prevention [14, 23].

Scholars of health communication engaged in an extensive discussion about the dominant paradigm of health; they discussed the importance of culture and urged incorporating the lay perceptions of cultural insiders in designing health communication [17, 24, 25]. In an initiative in 2002, the International Crisis and Emergency Risk Communication (CERC) model proposed community engagement as a strategy, to understand the cultural context of a particular community to build trust and also to identify residents’ perceptions to aid in developing educational messages during emergencies [20, 22]. The culture-centered approach is a bottom-up approach, explores the local meanings of a problem and how the cultural insiders of a target community interpret the risk of that problem [26]. This contextual understanding could inform practices related to disease transmission [27]. A culture-centered approach provides a platform to marginalized voices of the target community expressing their concerns and provides insights on how health decisions and meaning are negotiated within a given situation [28]. The goal of the health communicators in a culture-centered approach is to develop effective health messages that are responsive to the values and beliefs of the culture [26]. When addressing a health problem, health communicators need to understand these underlying cultural dimensions including the power dynamic and gender construction that are used to guide health intervention development [26] and could help to identify the inherent gaps, that need to be addressed in prevention initiatives [29]. However, health communication also needs to consider the possible limitation of lay knowledge on a specific issue which is new or not practiced in daily life [30]. Importantly, lay perceptions sometimes draw from a complex mixture and blending of biomedical knowledge, culture and livelihood experiences [29].

During outbreak investigations in Bangladesh, social scientists trained in anthropological approaches, initially explore local values, beliefs, social norms and care seeking practices related to the illness. Based on this contextual understanding, the outbreak investigation team further develops the messages and communication strategy to control the outbreak [15]. A defining characteristic of an acute high mortality infectious disease outbreak is the extremely short period available to develop a response. The difficult task for health communicators working as part of an outbreak response team [15] is to understand the gaps between the local knowledge and expert knowledge and to quickly develop a strategy that can effectively address these gaps [19]. Meeting the immediate health needs of the community may not always allow for thoroughly implementing all steps of an optimal approach, but rather requires streamlining practices using contextual understanding gained through the present and prior investigations in order to communicate relevant scientific information soon enough to be useful. Once the outbreak is over, for further or long-term prevention, it could ensure community engagement to frame a health problem and design for possible solutions. This paper illustrates this streamlining strategy we followed based on the contextual understanding during the short time frame in outbreak situation to communicate disease related information and prevention messages.

## Methods

### Study site

The outbreak occurred in two adjacent villages comprising a 2 km^2^ area within Bhanga sub-district in Faridpur District, in central Bangladesh. The residents had close social interactions; many of them were related through kinship.

### Study design, sampling and data collection

A team of three anthropologists and a sociologist conducted the anthropological investigation in January and February, 2010 during the outbreak. To make an initial connection with the residents, the outsider research team, who had different background with specialized education, needed to understand the societal context of the affected community. Because there was panic among community members, the research team first strove to develop a strong rapport with residents of the outbreak community to permit the investigation and as a basis for communicating prevention messages.

### Building rapport with community

To build rapport, we greeted the residents as we entered villages and initiated conversations with whomever we met on the village path. During our conversations, we listened to residents first and gave them an opportunity to express their views on the ongoing illness and hospital care, while we also responded to their queries. After basic rapport was established, we conducted an in-depth exploration of the outbreak and then communicated prevention messages. In the process of building rapport, when residents were sharing their concerns and queries, we learned that the local health authorities, who announced initial prevention messages, did not provide any details or causal explanations, nor did they respond to residents’ queries.

### Step 1: Exploring community beliefs and perceptions about the illness, care seeking and treatment practices

In this phase we collected information using several qualitative data collection tools. We conducted 10 informal discussions with community residents to understand the general perceptions of the disease outbreak and hospital care. Key informant interviews with two religious leaders and one religious scholar involved in ritual bathing of corpses of NiV case-patients and performing funeral ceremonies, allowed us to explore issues focused on funeral practices. We also carried out eight in-depth interviews with family members and friends who primarily cared for the NiV-infected cases during different stages of illness at home and in the hospital and also accompanied the dead body home from the hospital. Through these interviews we gathered exposure histories of infected cases, perceptions about the disease and transmission, care-seeking practices and the rationale for seeking particular types of care. Based on the interviews, we then recruited relatives and neighbours of the deceased cases who visited or cared for patients, performed ritual baths and funeral rites, and conducted four group discussions to cross-check their responses with those of other informants.

We conducted all the interviews and group discussions in a private setting or in a place preferred by the informants or participants. The team took detailed hand written notes and audio record most of the interviews and group discussions.

### Step 2: Communication of information and behaviour change messages

Based on the contextual understanding that we gained after exploring residents’ interpretations of the illness, we attempted to communicate the same prevention messages that announced previously, but using interactive communication instead of one-way announcement and employing more meaningful causal explanations. We arranged community group meetings to communicate information about NiV and how to prevent its transmission. To encourage many people to participate, we made house-to-house visits, and explained that we would respond to their queries related to this outbreak in the meeting. We also asked a team of community volunteers convened by the local health authority to help us in inviting residents to attend the meetings through household visits and loudspeaker announcements. Community residents selected the venue and time for each meeting.

**Fig. 1:**
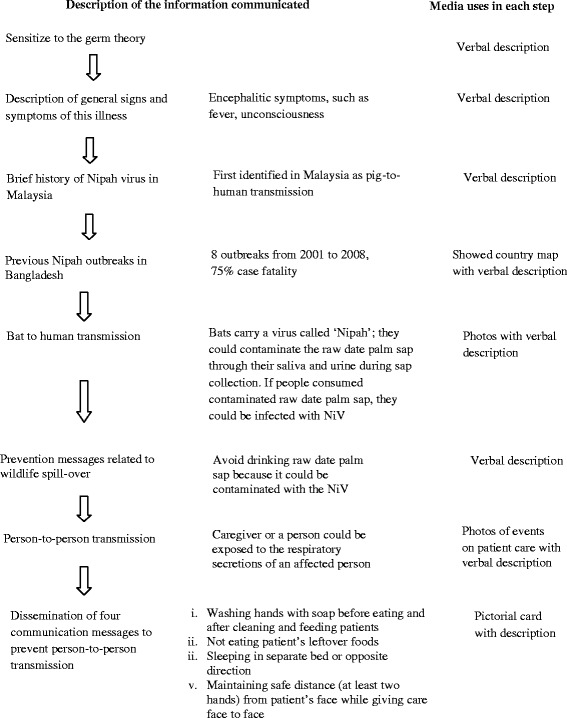
Flow chart of communicated information

**Fig. 2:**
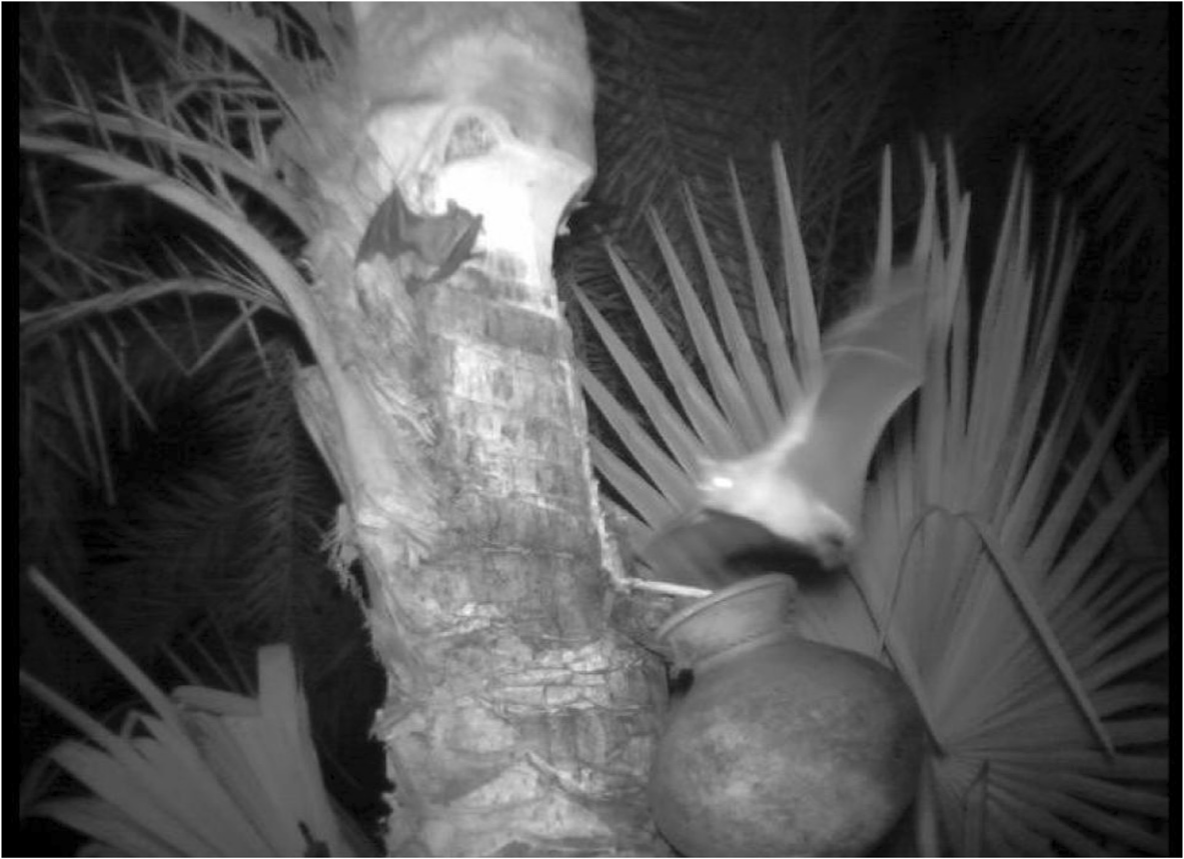
Infrared photo of bats drinking raw sap from pot during sap collection from date palm tree

We followed an interactive strategy for each meeting [31, 32]. In the meeting, we communicated the following information sequentially using lay language, relevant examples, and photographs:

To describe the biomedical model of an illness, we first explained to the participants how NiV spreads after entering the human body, how it affects the nervous system and other organs, and the signs and symptoms that would develop [3, 33, 34] (Fig. 1). We reminded participants using a local example about how cholera, an infectious disease familiar to the residents, was previously interpreted as a supernatural event before the invention and introduction of oral rehydration solution for treating cholera. People in the community at that time did not know how the germ (pathogen) caused cholera in humans. There were many myths about the cause of cholera, for instance, when an old woman known as “*cholera buri”* entered a village, she took away hundreds of lives and then stopped. However, people now understand it as a contagious disease and with the invention and popular use of oral rehydration solution, incidence of cholera or *patla paikhana-*related deaths decreased dramatically. We then described the brief history of the first identified NiV outbreak in Malaysia [35, 36]. We showed the participants a map of Bangladesh and highlighted affected districts where previous NiV outbreaks occurred from 2001 to 2008, listing the number of infected people and deaths [1, 2, 4, 5, 11]. After that, we showed infrared photos of bats drinking or licking raw sap from harvested date palm trees collected during research studies in Bangladesh, as well as showing bats trapped in the sap collection pot [13]. We explained that bats are the natural reservoir of this virus in Bangladesh and that bats could contaminate the raw date palm sap through their saliva and urine [1, 10, 11, 13, 37] (Fig. 2). Further, we explained that if people consumed this contaminated sap, they could be infected with NiV and develop encephalitic symptoms, such as fever and unconsciousness [3]. Using this causal explanation, we told them to avoid drinking raw date palm sap because it could be contaminated with NiV.

We then explained to the participants the typical care giving practices in Bangladesh, the potential risk of this behaviour, and how a person could be exposed to the respiratory secretions of an infected case [2, 14, 38]. Showing photos and pictorial cards, we described the four behaviour communication messages that could reduce risk of person-to-person transmission through contact with respiratory secretions: 1. washing hands with soap before eating and after cleaning and feeding patients, 2. not eating patient’s leftover foods, 3. sleeping in a separate bed or sleeping head to foot with the patient, and 4. always maintaining more than one hand’s distance between patient and caregiver faces. These messages were previously developed and piloted in a study with caregivers of patients hospitalized for pneumonia and meningo-encephalitis [39, 40]. (See flowchart for communicated information and messages in Fig. 1).

The meetings included a participatory question and answer session so that community residents could share their beliefs, perceptions, fears and concerns about the outbreak (See Additional file 1 for frequently asked questions and responses). Each of the four meetings lasted 90–120 min. In this step, two researchers facilitated the community meetings while the other two researchers took detailed notes of participants’ expressions, reactions, questions and responses. We also recorded all the meeting.

### Data analysis

We used standard approaches for the analysis and interpretation of qualitative data [41, 42]. We collected data in the native Bengali language**.** We expanded the notes for all the informal conversation, interviews and group discussions. In the time bound emergency situation, instead of doing verbatim transcription, we checked the audio records to fill in missing information. We developed a coding system to capture the main themes and concepts, and then collated responses. We combined and compared the data gathered from informal discussion, interview and group discussion and summarized ideas that addressed important themes [42] using the multiple respondents and data sources to cross-check for validity and ultimately enrich the interpretation of our data [43]. For step 2, we also counted the number of male and female participants in each meeting. The notes taken in the community meetings were analyzed using the same process as the in-depth interviews and group discussions in step 1.

## Results

### Spiritual beliefs and perceptions about Nipah virus and its transmission

Community residents had multiple explanations for the cause of the illness. They explained that supernatural causes related to the index case (the first identified case of this outbreak) and later close physical contact with the index case could have caused other people to become ill. The index case was a fisherman, who had been staying under a temporary shed beside a pond to catch fish three or four days prior to developing symptoms. According to family and neighbours, one night the index case saw three people coming towards him from the pond with blazing eyes. He was frightened, fainted and developed a fever the following morning. Some villagers also reported that both the index case and his wife were sleeping under the shed on that night which they considered an inappropriate setting for a husband and wife to sleep because of lack of privacy and this might be the cause of bad spirit. However, the wife denied this. As one villager said,

*Staying overnight in an open place is not good for a husband and wife; it might be a reason for this (frightened by the bad spirit) incident….*

This commonly shared story in the community spread fear that the illness was caused by a supernatural event related to the local fishing pond. Subsequently, when several persons developed similar symptoms, community members again interpreted the illnesses as *“asmani bala”,* or a crisis/hard time sent by Allah, to their area but did not know why Allah sent this (Allah is almighty in Islam and can give any hurdle at any time).

Families and neighbours of NiV-infected cases that our team attributed to person-to-person transmission believed that the effect of *kharap athta* (bad spirit) was the major cause of this illness. Some families of the deceased cases perceived that the illness could not be transmitted from one person to another and two caregivers reasoning that they consumed the leftover food of a NiV-infected case but did not get sick. Family and neighbours of another deceased case noted that he was only exposed when performing the ritual bath of the index case’s corpse but did not mention this exposure as a potential cause of his illness. In contrast, when they described their experiences, two cases’ families suggested that both supernatural forces and direct bodily transfer of the illness from the index case to their family members after close contact while caring for a patient, and washing a corpse or visiting the corpse could transmit illness.

Families of NiV-infected cases and community residents did not believe the initial messages provided by the local health authority, which said that drinking raw date palm sap could be the cause of the illness. Residents argued that they had been consuming this raw sap over generations without any adverse outcome. Thus, after receiving the initial prevention messages most of them continued the consumption of raw date palm sap. In an extreme event one deceased’s family continued to consume the raw sap even after the death of their father to prove that it was not the cause of his illness.

### Distrust of biomedical healthcare

During initial cases, families considered the illness as a “normal fever” and sought for biomedical treatment; but the failure of the biomedical treatment over the time to save lives, led to distrust. After the “supernatural event” with the index case, additional cases occurred, resulting in seven deaths spanning a three-week period from January 7^th^ to 28^th^, 2010. Simultaneously, several residents were also suffering from non-NiV associated febrile illness [6]. Initially, families of NiV-infected cases sought care from the unlicensed local village doctors (allopathic healer) and/or from a *fakir* (spiritual healers). When the families did not observe any improvement, all infected cases except the index case received treatment from private, licensed medical practitioners in their locality.

In a deteriorating condition when the NiV cases began talking incoherently or lost consciousness, families of five among seven cases took them to the local sub-district and tertiary-level government hospitals where they received treatment. The second infected case died at home the day after the index case’s death, after receiving treatment from local village doctors and also from a private licensed medical practitioner. Multiple fatalities in a short period of time resulted in families being afraid and reluctant to take other cases to the hospital. The seventh deceased case also received treatment from a private licensed medical practitioner and spiritual healers. This seventh case died at home; he became critically ill at night but the family did not consider taking him to the hospital because of transportation to the hospital was unavailable, and the previous five cases had not survived.

Every community resident we interviewed believed that the tertiary medical college hospital doctors intentionally killed these patients. They stated that the doctors were aware of this fatal disease, and tried to isolate these patients who came from the affected villages. Residents thought the doctors did not want the illness to spread within the hospital and into the wider community, so the doctors gave injections to the patients that killed them. One community resident whose son was suffering from a non-NiV febrile illness during our investigation and sought care from a private licensed medical practitioner explained,

*I am not interested in seeking treatment from the medical college hospital at all. We would rather see our dear one dying in front of our eyes, rather than sending him/her to the medical (college) hospital.*

The residents’ observation that the illness could not be cured by modern medical treatment strengthened their belief in the supernatural origin of the outbreak. Although no new case was identified after the death of the seventh case, the residents believed that the outbreak had not stopped because of ongoing non-NiV febrile illnesses that were occurring in the villages. They then called a religious healer who was the son of a religious leader in their village, but was living in Dhaka, the capital of Bangladesh. The healer told the villagers that an evil spirit had entered their villages and that this was the cause of the outbreak. By reciting Holy Quranic verses standing on the four corners of the villages, he assured the residents that he had driven away the spirit from the affected villages to other areas and from now the illness would stop affecting local people. The residents who were suffering from the fever also reported that after receiving treatment from the religious healer with blessed water to drink (*pani pora*) and with blowing (*fu dewa)* on patient’s body (pray with holy verses), they recovered. The villagers believed that by this process they become safe. Each household in the two villages paid the healer approximately US $ 0.4 to 2.00. In total, the healer earned approximately $300, twice the typical monthly income of a rural Bangladeshi family [44].

### Community responses: Uncertainties related to biomedical understandings of transmission

After building rapport, residents commented positively on our interactive approach that encouraged their active participation in a meeting. The immediate response from community residents reflected the positive impression of our home visits and interactions. Consistent with other residents one commented,

*They (the outbreak response team) came for our wellbeing. They came repeatedly to our community, and have come to our homes and invited us for the meetings; instead of our wellbeing what other interest might they have? …. they are doing a hard job only for our wellbeing; so we should attend the meeting.*

Around 100–150 residents among approximately 2,050 adult community population members participated in each of four meetings with the average ratio of males to females being 60:70.

During the community meetings, participants enquired that why did everyone who consume raw sap, not get the illness; why did only some of them affected; why it is happening now but not before (they had been drinking raw date palm sap for years without any problems)? These queries reflected uncertainties around the biomedical explanation of disease transmission, and also provided an opportunity to bridge biomedical and local understanding. In response, we explained that not all the bats shed this virus, only some of them are infected. Additionally, we explained three possible reasons for which one could not develop any illness after drinking raw sap; first, perhaps no bat came to drink the sap of the tree which you drunk; second, even if bats came and drunk the sap, we were not sure whether they were shedding the virus; third, some people might not be susceptible. (See Additional file 1).

Furthermore, after receiving the explanation about the route of transmission of NiV from bats to humans, along with the local example about how the understanding of how cholera is spread had shifted from a supernatural event to a germ as the cause, participants made connections. They reported that previous prevention messages had not convinced residents to stop drinking raw sap because they did not include information about bats being the source of this virus in the sap. Participants recalled that they had seen bats in and around the sap collection pot. They stated that the messages provided by us, combined with bats’ photo [Fig. 2], helped them understand how the virus could be transmitted in the sap. Participants then agreed that the outbreak was caused by a “virus” (NiV) which they had initially perceived as a supernatural force. In the meeting participants said,

*…(this illness) which we considered as ‘*asmani bala’ *(crisis/hard time given by Allah), you (the research team) call it virus. Now we have to agree that virus is the ‘bala’ (crisis to us) and ‘bala’ is the virus (to the team).*

Many participants also stated that they would abstain from raw date palm sap consumption.

During the meeting, participants described the collection of fluids from the back (cerebrospinal fluid) of some patients and administration of injections, which they believed caused the deaths of all the cases admitted in the hospital. They reported that the doctors carried out both of these medical procedures without explaining to the families why patients needed these (Additional file 1). In response, we explained the necessity of cerebrospinal fluid collection from infected cases in order to identify the virus that may be infecting the brain, and the participants accepted it.

While describing the four behaviour communication messages for preventing person-to-person transmission, referring to their prior query we explained that although many people may be exposed to an infected case, only a few of them may develop illness. In non-technical terms we replied that it might be due to differences in immune systems among humans or the strength of the virus or the level of exposure for which one might not develop illness after eating leftover food of a case patient. To avoid this transmission risk, every caregiver should follow the behaviours we had suggested. Respondents accepted messages about person-to-person transmission; one participant said,

*The patient may be my dear one but the disease is not. We have to follow what they (researchers) said to stay safe.*

### Culture-centric solutions

As we engaged with community responding their queries, we provided culturally credible explanations for several issues that depicted the culture-centered nature of risk management strategies. This emerged precisely because the research team included both ‘insider’ the community volunteer team and we as ‘outsider’ to the community. During our conversation in the meeting, we did not tell the residents to stop drinking raw date palm sap, rather we acknowledged and appreciated the cultural importance of this food, and provided ways by boiling in which they could still enjoy date palm sap, but minimize the risk.

We also highlighted the economic reality of this cultural practice of date palm sap collection and uncertainty of refraining people from sap consumption even if it banned. When participants described a local method of using *bana* (bamboo skirt, that covers both the tree trunk and the sap collection pot where sap is stored during collection), we explained that some of our researchers were testing the effectiveness of *bana* for safe sap collection and once they finish the research we could share the results.

We also did not tell the community to stop other cultural practices like ritual bathing of corpse, rather provided ways by covering mouth and washing hands-body with soap afterward that could minimize risk of transmission. Similarly, we explained why killing bats (as some participants suggested) to prevent NiV transmission would not be a good step since bats have ecological importance in pollination. (See details in Additional file 1).

## Discussion

During this sudden, fatal NiV outbreak, community residents blaming hospital doctors for patient deaths illustrated both the lack of community trust in the public healthcare system and the local interpretation of the illness. Our study findings suggest that the practice of not explaining the disease and the treatment or unfamiliar diagnostic procedures in the hospital risks amplifying community mistrust of government health service in the context of failure of biomedical treatment. The power dynamic between doctors and patients may encourage this type of interaction. In the social hierarchy of the dominant culture [45], rural patients are not usually allowed to express their voice or ask any treatment related technical process to doctors, who hold much higher social status. During this tense outbreak situation, developing rapport was crucial for building trust that could minimize the power distance between the affected residents and outsider research team or local health authority. Lack of trust and little hope for patient survival prompted residents to avoid seeking government hospital care. In this situation, the one-way communication approach adopted by the local health authority to the affected residents, an approach that lacked a meaningful causal explanation of transmission from bats to humans, and the missed opportunity for responding to residents’ logical queries was ineffective. A similar lack of trust was noted during previous Ebola epidemics in East Africa [23] or recent epidemics in West Africa which led affected patients to flee hospitals and hide cases [46].

We used a combined effort to build rapport including participatory approaches, such as home visits, interviews, group discussions and engaging insider volunteer team. The culture-centered approach [28] we followed created a comfortable environment for the residents to express their perception of the illness with the outbreak response team. During the emergency, there was a need to communicate certain expert information. By gaining residents’ trust and learning the outbreak context helped the team design an interactive communication strategy that facilitated the explanation of the biomedical paradigm of the disease outbreak and its’ transmission [27, 47]. The trust we built with the residents was also demonstrated by their active participation and responses in the community meetings.

Our study showed that residents primarily considered the illness as “normal fever” for which they seek biomedical treatment. Later, when they found medical treatment failed to save their dear one lives, they asked perfectly logical questions about the biomedical explanations. Their queries suggested that it is not simply spiritual beliefs that prevented people from understanding and believing science, rather uncertainties and failure of biomedical treatment contributed [19].

In developing countries, banning particular cultural practices is a common feature of health campaigns [48]. The team’s suggestion to avoid banning date palm sap and instead providing strategies to make it safer, represented the appreciative approach [49] towards local culture rather than “against the grain” of culture. This conflict between outsider’s professional advice and local cultural understanding has also been observed in Ebola outbreaks [23, 46]. In this NiV outbreak response, the information we provided during community meetings incorporated the local interpretation of the disease and its transmission ultimately enhanced residents’ understanding and credibility of biomedical information related to NiV. As the residents believed the transmission from person-to-person occurred from a sick person to another through direct body contact, not through any vehicle, such as secretions or saliva [50], our explanation persuaded them to accept germs as the cause of this disease and transmission.

However, the short time nature of the outbreak did not allow us to follow all the steps for an ideal culture-centered intervention, especially ensuring residents’ direct participations for developing messages or designing a communication strategy. In this short time frame, to design the intervention messages and strategy for this outbreak, we applied contextual learning from this outbreak as well as from the previous community based studies on NiV [14, 15]. We deployed an abbreviated approach to understand the community perspective, an approach that may have limited the depth and nuance of knowledge that would have resulted from a full in-depth investigation. Yet, we were able to provide insights that were available soon enough to be applicable to developing an emergency response. This approach could be applied in similar settings for responding to an emergency or outbreak situation. However, the communication strategy should be modified based on the particular communities’ knowledge, perceptions and practices regarding a particular outbreak.

One limitation of our communication effort is that we were not able to follow-up on the uptake of these behaviour messages since residents were not caring for any NiV-infected case nor were any newly infected cases identified after our communication of the messages. However, NiV surveillance staff reported that a patient from the affected community was admitted to the hospital as a suspected encephalitis patient immediately after the outbreak episode. Although he was later diagnosed with another disease, the caregiver of this patient followed the messages we suggested by sleeping in a separate bed and not eating the patient’s leftover food.

## Conclusions

During highly fatal outbreaks, instead of one-way communication, an interactive strategy communicated by a trained expert team, using lay language with supporting evidence, such as informative photos, can make the biomedical model of disease transmission and prevention messages credible to an affected community, even those who may initially invoke supernatural causal explanations.

Building rapport and trust with the residents of the affected community, is a prerequisite to understanding the local perception about the outbreak and a critical early step in the emergency response. Especially during an outbreak the central health authority should suggest that the local health authorities explain the necessity of treatment or diagnostic procedures to families while providing care. This may help to avoid miscommunication and potential mistrust between the affected communities and health professionals.

Our findings reinforce how a multidisciplinary team can work together to gain both biomedical and contextual understanding and to identify a culturally compelling prevention strategy during a crisis [24, 51, 52]. The communication team who delivers the messages needs to be technically skilled to understand the specific outbreak context in order to develop trust and identify dialogues that would be culturally credible to the affected community. Based on the local context, prevention messages need to be communicated during the outbreak using an interactive strategy with a logical causal explanation. Professional communicators or behavioural scientists may be better skilled than physicians for message delivery in such sensitive situations. Considering the specific context, expert teams and the local health authorities could work together to communicate these messages with the help of local community leaders or activists, such as union chairmen or members, or social activists of the village.

For recurrent outbreaks, such as NiV or Ebola, it would be useful to develop a set of communication materials a priori with participation of the local residents who have previous outbreak experience that could be deployed in future outbreak setting. Furthermore, follow-up assessments are needed to evaluate the effectiveness of this communication method.

## Abbreviations

CERC, International Crisis and Emergency Risk Communication; icddr,b, International Centre for Diarrhoeal Diseases Research, Bangladesh; IEDCR, Institute of Epidemiology, Disease Control and Research; NiV, Nipah virus

## Additional file

Additional file 1: Frequently asked questions and responses in the community meetings. This file includes list of questions that participants frequently asked to the facilitators (research team) during four meetings with community residents and the detailed responses the facilitators provided to the participants. This additional data will help the readers to get an overview of practical sessions how to deal the affected residents during an outbreak situation and respond to their concerns. The description included in the file also supported the basis of several points we presented under the “Results” section. (DOCX 24 kb)
