# Supplementary material for: It’s not only what you say, it’s also how you say it: communicating nipah virus prevention messages during an outbreak in Bangladesh
Source: BMC Public Health. 2016 Aug 5;16:726. doi: 10.1186/s12889-016-3416-z (PMC4974711; doi:10.1186/s12889-016-3416-z)
Supplement: Additional file 1: — Frequently asked questions and responses in the community meetings. This file includes list of questions that participants frequently asked to the facilitators (research team) during four meetings with community residents and the detailed responses the facilitators provided to the participants. This additional data will help the readers to get an overview of practical sessions how to deal the affected residents during an outbreak situation and respond to their concerns. The description included in the file also supported the basis of several points we presented under the “Results” section. (DOCX 24 kb) [file 12889_2016_3416_MOESM1_ESM.docx]

**Additional File 1**

**Frequently asked questions and responses in the community meetings ______________________________________________________________________________**

Title of the manuscript: It’s not only what you say, it’s also how you say it: Communicating Nipah virus prevention messages during an outbreak in Bangladesh

**______________________________________________________________________________**

***Q.*** *This disease has spread out through pigs in Malaysia but how it happened in our country?*

**Team response:** In response to this question we answered that, somehow *Nipah* virus has come into bats and bats are the natural reservoir of this virus. The virus itself does not affect bat, but when a bat that contains *Nipah* virus drink or leak the raw date palm sap during collection or eat any fruits, that virus can be transmitted into date palm sap or partially eaten fruits through body fluids of bat, like urine, feces or saliva. Afterward when people drink that virus contaminated raw sap, it can transmit into human body.

1. *Why the bat which bears this virus doesn’t develop this illness and die?*

**Team response:** Bats do not affect by this virus because of the existence of Nipah virus antibody in their body.

***Q.*** *If we consume date palm sap after boiling then will it cause any problem? How long we should boil them?*

**Team response:** No, it will not cause any problem. As far as we know it is safe to drink the sap after boiling.

1. *How can we stop drinking raw date palm sap?*

**Team response:** We said that many of us like the date palm sap very much. Before drinking raw sap, we should boil it. This virus is destroyed after boiling date palm sap.” At that time one male participant added, “We might drink raw date palm sap by using *bana* (bamboo net that covers both the tree trunk and the sap collection pot where sap is stored during sap collection).” Then we clarified that our research on *bana* is ongoing on; after finishing the research we can tell you briefly about the result.

1. *Can this sap collection practice be banned from Bangladesh?*

**Team response:** We said, “would we be stopped eating rice, if we find any harmful thing in rice? Many people in our country live on date palm sap harvesting and selling. So it may not possible to ban this practice from our country. Even if it is being banned, can you ensure that all of you will stop drinking it? Instead of this you can boil raw sap and use it to make sweet dishes or molasses and not take the raw sap.

1. *As this disease spreads out from bat so it is better to kill the bats.*

**Team response:** You know that each and everything of the nature is important. There is some important and positive role of bats in nature. Bats are very important for natural pollination; they eat harmful insects in the agricultural field. If we kill them, nature will be affected.

1. *Why they (hospital authority) kill people (patients) by pushing injections?*

**Team response:** No doctor wants to kill a patient. Every doctor has their professional ethics. If patients are taken to the hospital in that stage when virus already spread out all over the body, then the patient can die even at the hospital. For instance- a fewer amount of virus entered into my body. Then it will start reproduction and within a few days the body will be affected by a millions of viruses. But if we take the patient to hospital when the amount of virus is still less, we might save that patient through some supportive treatment. However, all the patients from your village were taken to the hospital not immediately after their onset of illness, rather after 3-5 days. So, the patient died after taken to the hospital.

In response to a similar question one of the volunteers (who helped the team to organize the meetings) responded that “Doctor does not want to kill a patient. All of you know that, XX vabi (wife of index case patient) was ill and at the first day of her illness I took her to the hospital. Now she is cured because of the treatment of those doctors. We took YY uncle (case patient) to the hospital at the last stage of his illness. Same thing happened for ZZ (case patient).”

***Q.*** *What are the symptoms of this disease?*

**Team response:** We responded that when the viruses enter into the human body, it attacks into human brain. The effected person could develop symptoms even after 11 days. The affected person could develop fever with headache; sometimes the patients cannot recognize people and have altered mental status *(bhul boke, manush chinte pare na),* or talk incoherently or lost his/her conscious level. Sometimes s/he cannot breathe properly; sometimes patients also have frothy discharged and convulsion (*khichuni*).

1. *How the disease transmitted from person to person?*

**Team response:** We explained that this disease is infectious. After developing symptoms, a patient could transmit the virus to other. During caring patient we should maintain some safety measures to prevent us from this disease. In our country when a person becomes ill, his/her family members are mainly responsible for taking care. Usually we feed our patient, sleep with patient and perform other physical care. This virus can transmit through patient’s saliva or froth or other body fluids. The caregivers usually experience a very close physical contact while caring. We often feed our patient or wipe up patient’s face or saliva and this virus could come into our hand with patient’s saliva. After that if our nose or mouth come contact with that contaminated hand, or we take food without washing hands with soap, this virus can enter into our body. Sometimes caregivers wipe up a patient's saliva and other respiratory secretions (sneeze, cough) with their own cloths, such as edge of saree or by a shared towel and commonly do not wash hands afterward. Later, if a caregiver comes in contact with those respiratory secretions or other bodily fluids from the cloths, same illness can be transmitted from a patient to caregiver.

***Q.*** *We have been drinking raw date palm sap for our life long but never developed any illness. So, why this illness is happening now?*

**Team response:** As we have said earlier that all the bats do not carry this virus, only some of them are the reservoirs. There can be three possible reasons for which you didn’t develop any illness even after drinking raw sap-

1. perhaps no bat came to drink the sap of the tree which you had drunk OR
2. even if bats came and drunk the sap, we are not sure whether they were carrying virus or not OR
3. disease resistance power (immunity) is not equal for all.

One of those could be a possible reason that even after drinking raw sap from the same pot, some people got illness while others did not.

***Q.*** *What measures are taken to prevent this disease?*

**Team response:** We should not drink raw date palm sap. If we want to drink date palm sap, we must boil it before drinking. We should not eat fruits, half eaten by bats or birds. Then we demonstrated the pictures of caring patients and explained them how to prevent person to person transmission.

***Q.*** *If a lactating mother develops this illness would her baby also develop the same illness by feeding the breastmilk?*

**Team response:** We still don’t know the answer but we are trying to understand and know about the disease as much as possible. So, we hope that soon we will come to know the answer of your question like many other unknown things about this disease.

***Q.*** *Many people fed rice to my uncle (Anwar, one of the case patients) and ate his leftover meal. Why they didn’t develop this illness?*

**Team response:** Again we explained the disease resistance power of the body.

1. *The gachi (sap collector) gave me the half amount of sap collected from my tree and mixed the other half in his own container. Later, why only the members of Mahji house (family of three cases) who bought the sap from that gachi and drank and developed the illness but not me?*

**Team response:** We explained that it might be possible there was no *nipah* virus in the date palm sap that collected from your tree. The *gachi* might collect the sap contaminated with *Nipah* virus from another date palm tree and mixed with the sap in his container and then sold it.

1. *Have the people also affected by Nipah virus who died in our area?*

**Team response:** We replied that many doctors have come here from the beginning of this outbreak. They collected blood from some of the patients who already died and their relatives. They also collected blood from bats and other animals. We have YY’s blood test report, which is *Nipah* positive. Others’ (control) blood test reports are negative. We did not have animals’ blood test report yet. We will inform you the result later.

1. *How this virus infection can be identified?*

**Team response:** We explained the procedure of collecting cerebrospinal fluid (CSF) as one of the diagnosis procedures and said that by examining this fluid, existence of virus can be confirmed.

1. *Isn’t there any medicine for this virus?*

**Team response:** Still there is no medicine for this virus, but if we could admit a patient in hospital in the early stage of the disease, doctor will try to save that patient by providing supportive treatment and s/he might be cured. We should try to save them.

***Q.*** *How the 35 people among 132 Nipah affected people were survived (in previous outbreaks)?*

**Team response:** We already described that how fatal disease NiV is. There is no such treatment of this illness has been discovered yet. Researchers are continuously working to understand more about this disease and its’ treatment. However, the patients who were survived in previous outbreaks because they might take to the hospital at the early stage of their illness. So far supportive treatments are given if patient could be taken to the hospital at the early stage of the illness. There is a chance or possibility to survive.

***Q.*** *How shall we perform funeral bathing? Should we wear plastic gloves? Is there any chance to get infected by touching the dead body or ill person with bare hands?*

**Team response:** We responded that definitely it is better if you could wear gloves. You can also cover your mouth and nose by a cloth during dead body's bathing. You should also wash your hands with soaps immediate after cleaning or washing the corpse.”

***Q.*** *Sometimes dead bodies also have frothy discharge. What should we do at that time?*

**Team response:** We should carefully wipe up the froth with a piece of cloth and then we must destroy (burn or buried) that cloth carefully without touching directly that froth. Immediate after that the person, who cleaned that froth, should wash his/her hands with soap.
